# Simultaneous acute Marchiafava–Bignami disease and central pontine myelinolysis: A case report of a challenging diagnosis

**DOI:** 10.1097/MD.0000000000009878

**Published:** 2018-02-23

**Authors:** Chun-Yi Tsai, Po-Kai Huang, Poyin Huang

**Affiliations:** a Department of Neurology, Kaohsiung Medical University Hospital, Kaohsiung Medical University, Kaohsiung, Taiwan; b Department of Pediatrics, E-Da Dachang Hospital; c Department of Neurology, Kaohsiung Municipal Hsiao-Kang Hospital, Kaohsiung Medical University, Kaohsiung, Taiwan; d Program in Translational Medicine, Kaohsiung Medical University and Academia Sinica; e Department of Neurology, Faculty of Medicine, College of Medicine, Kaohsiung Medical University, Kaohsiung, Taiwan.

**Keywords:** alcoholism, central pontine myelinolysis, Marchiafava–Bignami disease

## Abstract

**Rationale:**

Marchiafava–Bignami disease (MBD) is a rare disease characterized by demyelination of the corpus callosum. It is most commonly seen in patients with chronic alcoholism. The clinical diagnosis of MBD can be difficult due to its nonspecific manifestation. Central pontine myelinolysis (CPM) occurs mostly as a complication of severe and prolonged hyponatremia, especially when corrected too rapidly. However, CPM can be associated with chronic alcoholism and its clinical presentation can be heterogeneous. Because both MBD and CPM can have fatal outcomes, early recognition and treatment can result in a better prognosis. We present a very rare case of simultaneous acute Marchiafava–Bignami disease and central pontine myelinolysis in a patient with chronic alcoholism who was diagnosed unexpectedly using brain magnetic resonance imaging and improved after proper treatment.

**Patient concerns:**

We presented a case of a 39-year-old patient who visited the hospital with general weakness and an altered neurologic condition after a week of vomiting.

**Diagnosis:**

The patient was diagnosed with simultaneous acute Marchiafava–Bignami disease and central pontine myelinolysis using brain magnetic resonance imaging.

**Intervention:**

Administration of a high dose of thiamine.

**Outcomes:**

The neurologic signs improved after a week of thiamine administration.

**Lessons:**

This case suggests that Marchiafava–Bignami disease and central pontine myelinolysis might have a common pathogenesis, and brain magnetic resonance imaging is of crucial importance in chronic alcoholic patients presenting with nonspecific neurological deterioration. The appropriate administration of thiamine may prevent poor outcomes.

## 1 Introduction

Marchiafava–Bignami disease (MBD) characterized by demyelination and necrosis of the corpus callosum is a rare but fatal condition mainly associated with alcoholism and malnutrition. The clinical manifestation of MBD is nonspecific with a wide variation, including split-brain syndrome, difficulty walking, para- or tetraparesis, altered mental status, seizure, and even coma or death.^[1–3]^ The remarkable recovery of clinical condition and the resolution of imaging abnormalities have been reported in patients receiving thiamine treatment.^[4–6]^ Central pontine myelinolysis (CPM) also has heterogeneous clinical presentation including confusion, quadriplegia, pseudobulbar palsy, and coma. CPM is mostly associated with the rapid correction of hyponatremia but could be caused by alcoholism in rare conditions. Herein, we present a rare case of a chronic alcoholic patient who suffered from MBD and CPM simultaneously, which suggests that MBD and CPM might have a common pathogenesis or metabolic deficiency.

## 2 Case report

**Figure 1:**
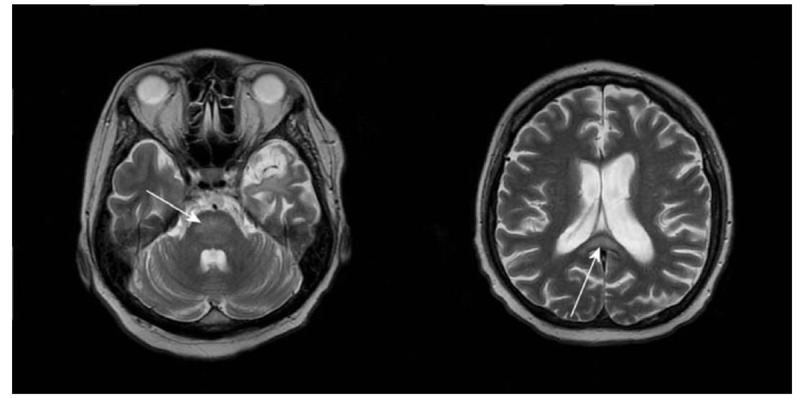
Brain MRI (axial). T2 hyperintensity (white arrow) of the central pons (left) and the splenium of the corpus callosum (right). MRI = magnetic resonance imaging.

A 39-year-old male presented to our emergency department due to the acute onset of gait disturbance and deterioration of mental status. He had altered mental status with confusion and bradyphrenia for years. Concomitantly, he had an ataxic gait but could walk slowly and ascend stairs independently. His family noted that his gait had changed in the preceding week. He could not walk or ascend stairs by himself. He also had bilateral hand tremors and his cognitive function had deteriorated. These symptoms and signs progressed within one week. Neurological examination showed bilateral lower limb weakness without sensory impairment. The muscle power of the bilateral lower limbs was only grade 4. There was normal muscle tone without spasticity. Impaired cerebellar signs, such as dysmetria and truncal ataxia, were also noted. He had no autonomic systems involved. Reviewing his history, his family reported that he had consumed a daily average of 1000 to 2000 mL of rice alcohol for an unknown long period. He had also consumed amphetamines for an unknown period but had quit. The patient was in poor physical condition and seemed malnourished. He had chronic diarrhea and frequent vomiting in the preceding week. He had no history of head trauma, recent fever, or infection. There was no similar family. Routine laboratory tests including electrolytes, liver function, ammonia level, vitamin B12, and folic acid were all unremarkable. No hyponatremia was noted. A brain computed tomography (CT) showed unremarkable hypodensity at the central pons and diffuse cortical and cerebellar atrophy. His brain magnetic resonance imaging (MRI) showed hyperintensity on the T2-weighted image in the splenium of the corpus callosum and central pons while there was no evidence of acute infarction on the diffusion-weighted image (Fig. 1). His EEG was in line with normal cerebral cortical function without detectable epileptogenicity. Simultaneous Marchiafava–Bignami disease, central pontine myelinolysis, and Wernicke's encephalopathy were diagnosed based on his history of chronic alcoholism and specific brain MRI findings. High-dose vitamin B complex including 1000 mg/day of thiamine was administered intravenously. After treatment, the muscle power of his bilateral lower limbs kept improving. Upon discharge, his muscle power was grade 4+. His ataxia was also improving. A fatal outcome was avoided. However, his cognitive impairment remained the same. He did not report any side effects of thiamine administration. The patient was lost to follow-up; thus, further brain MRIs could not be arranged for the evaluation of the therapeutic effects.

We did not have patient consent because there were no identifiable patient characteristics in this case report.

## 3 Discussion

MBD characterized by demyelination and necrosis of the corpus callosum is a rare but fatal condition mainly associated with alcoholism and malnutrition. The clinical manifestation of MBD is nonspecific with a wide variation, including acute onset split-brain syndrome, difficulty walking, para- or tetraparesis, altered mental status, seizure, and even coma or death.^[1–3]^ The remarkable recovery of clinical condition and the resolution of imaging abnormalities have been reported in patients receiving thiamine treatment. Early diagnosis and thiamine administration may result in a better prognosis.^[4–6]^ Thiamine deficiency is often seen in patients with alcoholism, malnutrition, and frequent vomiting. The exact mechanism of how thiamine treatment may be effective for MBD remains unclear.^[1]^

CPM also has a heterogeneous clinical presentation including acute onset confusion, quadriplegia, pseudobulbar palsy, and coma. CPM is mostly associated with the rapid correction of hyponatremia. However, CPM could be caused by chronic alcoholism without the rapid correction of hyponatremia.^[2,6]^

The concomitant presence of CPM and improvement with thiamine therapy suggest a common pathogenesis or metabolic deficiency. Thiamine might play an important role in the metabolic pathway. Thiamine is a cofactor of some critical enzymes in the Krebs cycle.^[4–6]^ Thiamine insufficiency weakens the cells’ ability to regulate the osmotic gradients and may result in cytotoxic edema. It has been proposed that oligodendrocytes, typically within the white matter, may be vulnerable to osmotic stress when located near gray matter. The coexistence of MBD and CPM could be explained by the osmotic changes that affect the regions mixed with gray and white matter elements.^[2,5]^ Thus, chronic alcoholism and malnutrition can cause MBD or CPM. However, it is very rare to have simultaneous MBD and CPM. Since both MBD and CPM may have nonspecific clinical manifestations, it would be rather difficult to diagnose simultaneous MBD and CPM by clinical manifestations alone. A brain MRI is of crucial importance for a prompt and correct diagnosis. The presentation of MBD on brain MRI are low signal density on T1-weighted images, high signal density on T2-weighted images, and fluid-attenuated inversion recovery located at the corpus callosum. The image findings of CPM are low density on CT and altered signal on MRI in the central pons.^[1–3]^

Our patient was alcoholic with malnutrition and chronic diarrhea. His cognitive presentation and chronic ataxic gait met the diagnosis of Wernicke–Korsakoff encephalopathy. However, his acute worsening neurological condition may have been the result of MBD and CPM. Although he had no hyponatremia, frequent vomiting in the preceding week may have been the aggravating factor. Thiamine deficiency and frequent vomiting change the osmotic gradient and damage vulnerable areas. In this case, the clinical manifestation was nonspecific. A brain MRI is of crucial importance for the diagnosis of MBD and CPM, leading to prompt treatment with thiamine that could be life-saving. This case suggests that MBD and CPM might have a common pathogenesis/metabolic deficiency and a brain MRI is of crucial importance for chronic alcoholic patients presenting with nonspecific neurological deterioration.

## Author contributions

C-Y: acquisition of data, drafting the manuscript.

PH: acquisition of data, drafting the manuscript

PH: corresponding author, design and coordination of the study, interpretation of data, drafting the manuscript.
